# Comparison of Liposomal Bupivacaine for Single‐Injection Versus Dual‐Injection Thoracic Paravertebral Block on Analgesic Effect After Dual‐Microport Thoracoscopic Lobectomy: A Prospective Randomized Controlled Trial

**DOI:** 10.1155/prm/8822825

**Published:** 2026-07-02

**Authors:** Yue Shang, Wenlun Zhong, Jiameng Wang, Chenyang Wang, Li Sun, Changjun Gao

**Affiliations:** ^1^ Department of Anesthesiology, Tangdu Hospital, the Fourth Military Medical University, Xi’an, Shaanxi, China, fmmu.edu.cn; ^2^ Graduate School, Xi’an Medical University, Xi’an, Shaanxi, China, xjtu.edu.cn; ^3^ Graduate School, Yan’an University, Yan’an, Shaanxi, China, yau.edu.cn

**Keywords:** dual-injection thoracic paravertebral block, dual-microport thoracoscopic lobectomy, liposomal bupivacaine, postoperative analgesia, single-injection thoracic paravertebral block

## Abstract

**Background:**

The optimal blockade strategy for thoracic paravertebral block (TPVB) with liposomal bupivacaine (LB) in dual‐microport thoracoscopic lobectomy remains unclear. This study evaluated the analgesic efficacy of single‐injection versus dual‐injection LB in TPVB.

**Methods:**

This prospective randomized controlled trial enrolled patients undergoing elective dual‐port thoracoscopic surgery from June to September 2025. Participants were randomly assigned to either a single‐injection group (Group S) or a dual‐injection group (Group D). Under ultrasound guidance, Group S received a single 20 mL (266 mg) injection of LB at the thoracic vertebrae 4‐5, while Group D received two separate 10 mL (133 mg) injections at the thoracic vertebrae 3‐4 and 6‐7. The primary outcome was the area under the curve (AUC) of the numerical rating scale (NRS) scores during movement over 72 h postoperatively.

**Results:**

A total of 92 patients were included in this study, with 46 patients allocated to each group. The mean AUC (SD) of NRS scores during movement at 72 h postoperatively for Groups S and D was 148.83 (80.18) and 201.52 (111.99) NRS/h, respectively, with a mean difference of 52.69 (95% CI 12.4 to 93.0, *p* = 0.011). The incidence of chronic pain at 3 months postoperatively was 19.6% in Group S and 21.7% in Group D (*p* = 0.798). Generalized estimating equation analysis showed that at rest, Group S had lower NRS scores than Group D at 48 h postoperatively (95% CI ‐1.4 to −0.2, *p* = 0.044). During movement, the median NRS scores (IQR) at 24 h for Groups S and D were 3.0 (2.0, 3.0) and 3.0 (2.0, 5.0), respectively, with *p* = 0.009; at 48 h, the median NRS scores (IQR) were 2.0 (1.0, 3.0) and 2.5 (2.0, 5.0), respectively, with *p* = 0.014. The median block duration (IQR) for Groups S and D was 7.00 (6.00, 8.00) and 11.00 (9.75, 12.00), respectively, with *p* < 0.01; the median hospital stay (IQR) was 4.00 (3.00, 4.25) and 4.00 (4.00, 5.00), respectively, with *p* = 0.045.

**Conclusion:**

Single‐injection LB in TPVB provides comparable or superior analgesic efficacy while significantly reducing the risk of postoperative complications, making it a preferred analgesic strategy.

**Trial Registration:** Chinese Registry of Clinical Trials: ChiCTR2500104841

## 1. Introduction

Compared to traditional single‐port or multiport thoracoscopic lobectomy, dual‐microport thoracoscopic lobectomy significantly minimizes tissue damage by requiring only three small incisions. However, previous studies have shown that, even with dual‐microport thoracoscopic lobectomy, up to 50% of patients still experience moderate‐to‐severe pain during the early postoperative period, the intensity and duration of which are often underestimated [1]. Inadequate management of acute postoperative pain can directly impair patients’ ability to cough and expectorate, thereby increasing the risks of pulmonary infections and atelectasis, prolonging hospital stays, and potentially leading to chronic postsurgical pain (CPSP) if the pain persists beyond 3 months [2, 3].

PROSPECT guidelines recommend thoracic paravertebral block (TPVB) as the primary regional anesthesia technique for managing postoperative pain following thoracoscopic lobectomy [4]. However, ropivacaine and bupivacaine, commonly used in TPVB, provide analgesia for only 6–12 h with a single injection, which is insufficient to cover the peak pain period in the first 72 h postoperatively, necessitating reliance on patient‐controlled intravenous analgesia (PCIA) for prolonged pain relief [5–7]. Liposomal bupivacaine (LB), an extended‐release local anesthetic, functions as a depot at the injection site, providing slow and sustained drug release, offering prolonged analgesia for up to 72 h after a single administration [8]. Its safety profile has been confirmed in several large clinical trials and is especially recommended for local infiltration at the surgical site and peripheral nerve blocks [9–11]. In thoracic surgery, studies on the analgesic efficacy of LB in serratus anterior plane blocks and intercostal nerve blocks have demonstrated its ability to extend the duration of analgesia and enhance early postoperative recovery quality [12–14]. However, evidence regarding LB’s use in TPVB specifically within the context of dual‐microport thoracoscopy is limited. Notably, the optimal injection strategy—whether a concentrated single‐injection or a distributed dual‐injection approach—has yet to be addressed in prospective randomized controlled trials. Given the depot‐forming property of LB, a single, higher concentration injection may produce a more sustained release and sufficient longitudinal spread to cover the T3‐T7 dermatomes, whereas dividing the same total volume might reduce peak local concentration and compromise the prolonged analgesic effect. Additionally, our pilot study data (20 patients, 10 per group) showed that the single‐injection group had a lower mean area under the curve (AUC) of movement numerical rating scale (NRS) scores over 72 h compared to the dual‐injection group, suggesting a potential analgesic advantage. However, given the small sample size of the pilot study, these findings were preliminary and required confirmation in a larger trial.

Thus, this study aimed to compare the analgesic efficacy of single‐injection versus dual‐injection LB in TPVB for patients undergoing dual‐microport thoracoscopic lobectomy. It is hypothesized that the single‐injection method would provide superior analgesia within the first 72 h postoperatively compared to the dual‐injection method, significantly reducing postoperative pain scores, rescue opioid consumption, and length of hospital stay.

## 2. Methods

### 2.1. Trial Design

This prospective, randomized, controlled, single‐center trial was approved by the Ethics Committee of Tangdu Hospital, Air Force Medical University on June 14, 2025 (Approval No. K202506‐14). This trial adhered to the CONSORT guidelines.

### 2.2. Participants

Eligible participants met the following criteria: scheduled for dual‐microport thoracoscopic lobectomy (wedge resection, segmentectomy, or lobectomy) under general anesthesia; aged ≥ 18 years; body mass index (BMI) between 18 and 30 kg/m^2^; American Society of Anesthesiologists (ASA) Physical Status I or II; and voluntary written informed consent.

Exclusion criteria included refusal to participate; communication barriers or inability to cooperate with the study (e.g., language comprehension disorders, psychiatric conditions, epilepsy, Parkinson’s disease, or myasthenia gravis); history of alcohol, analgesic, or substance abuse; known allergies to study medications; chronic pain in the chest or back lasting ≥ 3 months; prior thoracic surgery; or other conditions deemed unsuitable by the attending physician or investigator.

Withdrawal criteria included intraoperative massive hemorrhage (blood loss > 800 mL); conversion to open thoracotomy; loss to follow‐up (e.g., due to death or refusal of telephone follow‐up); or the need for subsequent thoracoscopic or open thoracic surgery.

### 2.3. Randomization and Allocation

Research personnel were divided into two groups: One, blinded to group allocation, was responsible for preoperative visits and postoperative follow‐up, while the other group managed randomization, drug administration, and preparation of the trial medications. We blinded the patients, surgeons, and follow‐up assessors. The anesthesiologists administering the nerve blocks remained unblinded. Randomization was performed using a computer‐generated list, and patients were assigned to either the single‐injection group (Group S) or the dual‐injection group (Group D). Allocation was concealed using sealed envelopes. In Group S, 20 mL of 1.33% LB (266 mg) was injected at thoracic vertebrae 4‐5, while in Group D, 10 mL of the same solution (totaling 20 mL and 266 mg) was injected at thoracic vertebrae 3‐4 and 6‐7.

### 2.4. Interventions

Preoperative assessments were conducted for all patients. Upon entering the operating room, routine vital sign monitoring was initiated. General anesthesia was induced with midazolam (0.03 mg/kg), propofol (2 mg/kg), sufentanil (0.5 μg/kg), and rocuronium (0.6 mg/kg). After induction, patients were positioned laterally, and under ultrasound guidance, the thoracic paravertebral space was identified. A 20 G, 10 cm needle was advanced into the space, and after confirming no aspiration of blood, 20 mL of local anesthetic was injected. Intraoperative anesthesia was maintained using total intravenous anesthesia with remifentanil (0.05–2 μg/kg/h), propofol (4–6 mg/kg/h), and intermittent boluses of rocuronium. A PCIA pump was initiated immediately postsurgery, configured with a 100 mL solution containing sufentanil (100 μg) and tropisetron (5 mg), set at a continuous infusion rate of 2.5 mL/h, a bolus dose of 3 mL, and a lockout interval of 15 min. Rescue analgesia with 100 mg of tramadol was administered intravenously as a single dose whenever the NRS score reached ≥ 4 points.

No additional intraoperative sufentanil was administered beyond the induction dose. Perioperative administration of paracetamol, nonsteroidal anti‐inflammatory drugs, or dexamethasone was not part of the routine protocol, and none of the enrolled patients received these medications during the study period, as confirmed by medical record review. Thus, the analgesic outcomes were not confounded by these co‐interventions.

### 2.5. Outcome Measures

Preoperative Data: Age, gender, ASA physical status classification, BMI, smoking and alcohol history, pain status, duration of illness, and lesion location.

Intraoperative Data: Resected lung tissue, lung nodule diameter, pathological type, operative time, anesthesia time, intraoperative fluid balance (including blood loss, transfusion volume, fluid intake, and output), use of other medications, and intraoperative blood pressure fluctuations.

Postoperative Data: Pain scores at rest and during movement (coughing) assessed using the NRS scale at 6 h, 24 h, 48 h, 72 h, and 3 months postoperatively; consumption of rescue opioid medication (tramadol) on Postoperative Days 1–7; incidence of postoperative adverse reactions; postoperative pulmonary complications (assessed *via* CT scans within 7 days postoperation); length of postoperative hospital stay; number and duration of drainage tube placements; duration of stay in the postanesthesia care unit (PACU); number of effective and total PCIA attempts; and TPVB procedure duration.

The primary endpoint was the AUC of the NRS scores during movement over 72 h postoperatively. Secondary outcomes included the AUC of resting NRS scores within 72 h postoperatively, the incidence of CPSP at 3 months, total and effective PCIA attempts within 48 h postsurgery, TPVB procedure duration, length of hospital stay, rescue analgesia requirements within 7 days postoperatively, and the incidence of postoperative adverse events and pulmonary complications.

The highest NRS score recorded within the first 72 h postoperatively served as the criterion for assessing acute postoperative pain intensity, where 0 indicated no pain, 1–3 represented mild pain, 4–6 indicated moderate pain, and 7–10 represented severe pain [15]. CPSP was defined as an NRS score > 0 reported by the patient at 3 months postoperatively [16, 17].

### 2.6. Sample Size Calculation

Based on pilot study data from 20 patients (10 in Group S and 10 in Group D), the mean AUC for pain during movement over 72 h was 144.7 ± 34.8 for Group S and 184.9 ± 37.6 for Group D, yielding a difference of 40.2. The pooled standard deviation was 36.23. With a significance level (*α*) of 0.05 and a power (1‐β) of 0.8, the calculated sample size required was 82 patients. Considering a 10% anticipated dropout rate, a total of 92 patients were planned for enrollment, with 46 patients allocated to each group.

### 2.7. Statistical Analysis

Data entry and verification were performed using Excel, while all statistical analyses were conducted with SPSS software (Version 27.0). All tests were two‐sided, and a *p* value of less than 0.05 was considered statistically significant. The normality of continuous variables was assessed using the Kolmogorov–Smirnov test. Continuous data with a normal distribution are presented as mean ± standard deviation and were compared between groups using the independent‐samples *t*‐test; within‐group comparisons were made using repeated‐measures analysis of variance. Continuous data not following a normal distribution are presented as median (interquartile range [IQR]) and were compared between groups using the Mann–Whitney *U-*test. Categorical data are expressed as frequency (percentage) and were compared between groups using the chi‐square test or Fisher’s exact test, as appropriate.

For the longitudinal analysis of postoperative pain scores, a generalized estimating equation (GEE) model was used to account for the correlation of repeated‐measures data. Missing data were handled using the following approach: First, Little’s Missing Completely at Random (MCAR) test was performed to verify that the data met the MCAR assumption. A stratified handling strategy was then applied: Variables with missing data < 5% were handled by listwise deletion, while scattered missing values in continuous variables were imputed using the arithmetic mean. To ensure the robustness of the findings, all statistical models were run on both the original complete case dataset and the imputed dataset. The high consistency of results across these analyses validated the reliability of the conclusions.

## 3. Results

**FIGURE 1:**
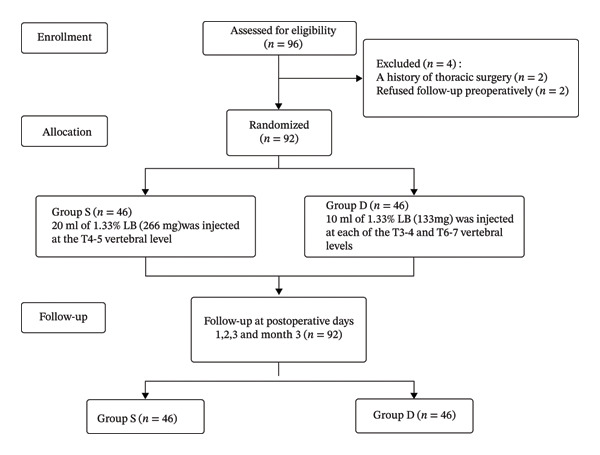
Study flowchart.

The first patient was enrolled on June 25, 2025, and the last patient was enrolled on September 13, 2025. A total of 96 patients were assessed for eligibility. Four patients were excluded: two due to a history of thoracic surgery and two due to refusal to participate in follow‐up preoperatively. As a result, 92 patients were randomized into Group S or Group D and completed the trial (Figure 1).

### 3.1. Analysis of Baseline Characteristics

Baseline characteristics were evenly distributed between the two groups, with no significant differences observed (Table 1).

**TABLE 1:** Baseline and surgical characteristics.

Characteristic	Group D (*N* = 46)	Group S (*N* = 46)	*p*
Age (years)	58.02 ± 11.36	55.67 ± 11.87	0.408
Gender, *n* (%)			0.674
Male	21 (45.7%)	19 (41.3%)	
Female	25 (54.3%)	27 (58.7%)	
BMI (kg/m^2^)	23.35 ± 2.38	23.78 ± 3.07	0.409
ASA physical status, *n* (%)			1.000
I	2 (4.3%)	3 (6.5%)	
II	44 (95.7%)	43 (93.5%)	
Smoking history, *n* (%)			0.509
Never smoker	30 (65.2%)	32 (69.6%)	
Current smoker	6 (13.0%)	8 (17.4%)	
Former smoker	10 (21.7%)	6 (13.0%)	
Alcohol history, *n* (%)			0.165
Never drinker	37 (80.4%)	37 (80.4%)	
Current drinker	9 (19.6%)	6 (13.0%)	
Former drinker	0 (0%)	3 (6.5%)	
History of surgery, *n* (%)	24 (52.2%)	26 (56.5%)	0.675
Hypertension, *n* (%)	11 (23.9%)	14 (30.4%)	0.482
Coronary heart disease, *n* (%)	3 (6.5%)	2 (4.3%)	1.000
Diabetes mellitus, *n* (%)	4 (8.7%)	5 (10.9%)	1.000
Duration of illness (months)	3.00 (1.00, 15.00)	4.50 (1.00, 24.00)	0.388
Lesion location, *n* (%)			0.388
Left	19 (41.3%)	15 (32.6%)	
Right	27 (58.7%)	31 (67.4%)	
Pathological type, *n* (%)			0.714
Malignant	41 (89.1%)	43 (93.5%)	
Benign	5 (10.9%)	3 (6.5%)	
Resected lung tissue, *n* (%)			0.616
Lobectomy	17 (37.0%)	18 (39.1%)	
Segmentectomy	14 (30.4%)	10 (21.7%)	
Wedge resection	15 (32.6%)	18 (39.1%)	
Operative time (min)	97.96 ± 45.95	99.00 ± 38.33	0.708
Anesthesia time (min)	128.54 ± 48.26	129.74 ± 41.43	0.764
Blood loss (mL)	20.00 (10.00, 50.00)	20.00 (20.00, 50.00)	0.396

Abbreviations: ASA, American Society of Anesthesiologists; BMI, body mass index.

### 3.2. The AUC of NRS Scores at Rest and During Movement Within 72 Hours Postoperatively

**FIGURE 2:**
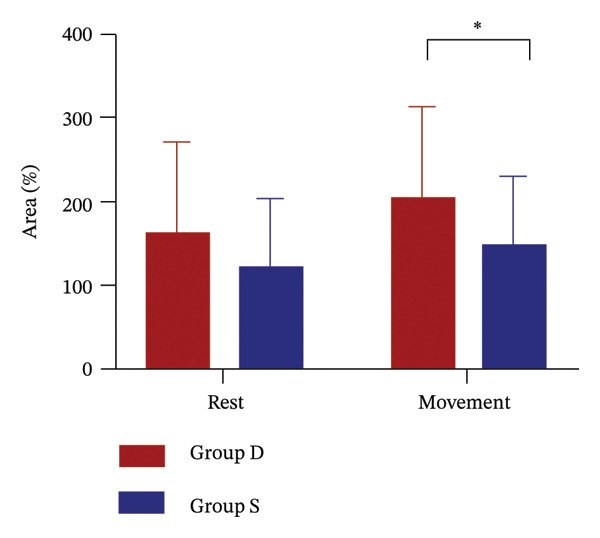
Boxplot comparing the AUC of NRS scores at rest and during movement.

During movement, the AUC of NRS scores was significantly higher in Group D compared to Group S (201.52 ± 111.99 versus 148.83 ± 80.18, respectively; *p* = 0.011). At rest, the AUC of NRS scores was 160.76 ± 111.71 in Group D and 120.98 ± 82.04 in Group S (*p* = 0.055), with no statistically significant difference between the groups. These results suggest that, compared to the dual‐injection method, the single‐injection method significantly reduced the overall pain burden during movement in the first 72 h postoperatively. The improvement was more pronounced during movement (a 24.8% reduction in Group S compared to Group D) than at rest (a 19.7% reduction) (Figure 2).

### 3.3. Postoperative Pain Scores

During the first 72 h postoperatively, the distribution of pain severity was as follows: At rest, 37.0% (17/46) of patients in Group S and 39.1% (18/46) in Group D experienced moderate pain. Severe pain was reported by 10.9% (5/46) of patients in Group D, while none in Group S reported severe pain. During movement, moderate pain was experienced by 45.7% (21/46) of Group S and 50.0% (23/46) of Group D. Severe pain was reported by 13.0% (6/46) in Group D, while no severe pain was reported in Group S. GEE analysis revealed that pain scores during movement were significantly lower in Group S compared to Group D at 24 h postoperatively (*β* = −0.9, 95% CI: −1.5 to −0.2; *p* = 0.009), and this advantage persisted at 48 h (*β* = −0.8, 95% CI: −1.5 to −0.2; *p* = 0.014). At rest, NRS scores were lower in Group S at 48 h, but no statistically significant differences were found at other time points (Table 2). The incidence of CPSP was 19.6% (9/46) in Group S and 21.7% (10/46) in Group D (*p* = 0.798). These results indicate that single‐injection blockade provides superior dynamic analgesia during the critical 24–48‐h postoperative recovery period, which is more aligned with the clinical need for early ambulation in thoracoscopic lobectomy patients.

**TABLE 2:** Comparison of NRS scores between the two groups.

Variables	Group D (*N* = 46)	Group S (*N* = 46)	β	95% CI	*p*
Rest					
Preoperative	0	0	Reference	—	
6 h postoperatively	3.0 (0.0, 5.0)	2.0 (0.75, 4.0)	−0.6	−1.5, 0.3	0.220
24 h postoperatively	2.0 (0.75, 3.0)	2.0 (1.0, 3.0)	−0.5	−1.1, 0.2	0.150
48 h postoperatively	2.0 (1.0, 4.0)	1.0 (0.0, 3.0)	−0.7	−1.4, −0.1	0.044
72 h postoperatively	1.0 (0.0, 2.25)	0.5 (0.0, 2.0)	−0.5	−1.1, 0.1	0.104
3 months postoperatively	0 (0, 0)	0 (0, 0)	−0.1	−0.4, 0.2	0.455
Movement					
Preoperative	0	0	Reference	—	
6 h postoperatively	3.0 (0.0, 5.25)	2.0 (0.75, 4.0)	−0.6	−1.6, 0.3	0.181
24 h postoperatively	3.0 (2.0, 5.0)	3.0 (2.0, 3.0)	−0.9	−1.5, −0.2	0.009
48 h postoperatively	2.5 (2.0, 5.0)	2.0 (1.0, 3.0)	−0.8	−1.5, −0.2	0.014
72 h postoperatively	2.0 (0.75, 3.0)	1.0 (0.0, 3.0)	−0.5	−1.1, 0.1	0.076
3 months postoperatively	0 (0, 0)	0 (0, 0)	−0.2	−0.6, 0.3	0.489

Abbreviation: CI, confidence interval.

### 3.4. Postoperative Pulmonary Complications and Adverse Reactions

The TPVB procedure duration was significantly longer in Group D compared to Group S (*p* < 0.01). No statistically significant differences were observed in the overall incidence of postoperative pulmonary complications or adverse reactions between the two groups, and no serious adverse events, such as respiratory depression or local anesthetic systemic toxicity (LAST), occurred in either group (Table 3).

**TABLE 3:** Postoperative pulmonary complications and adverse reactions.

Indicator	Group D (*N* = 46)	Group S (*N* = 46)	*p*
Pulmonary complications, *n* (%)			
Hydropneumothorax	31 (67.4%)	36 (78.3%)	0.241
Exudative changes	29 (63.0%)	32 (69.6%)	0.508
Pneumonia	12 (26.1%)	17 (37.0%)	0.262
Pleural effusion	12 (26.1%)	18 (39.1%)	0.182
Atelectasis	8 (17.4%)	10 (21.7%)	0.599
Subcutaneous emphysema	5 (10.9%)	7 (15.2%)	0.536
Adverse reactions, *n* (%)			
Nausea and vomiting	6 (13.0%)	3 (6.5%)	0.485
Dizziness	2 (4.3%)	0 (0%)	0.495
Pruritus	1 (2.2%)	0 (0%)	1.000
Respiratory depression	0 (0%)	0 (0%)	1.000
LAST	0 (0%)	0 (0%)	1.000
TPVB procedure duration (min)	11.0 (9.75, 12.00)	7.00 (6.00, 8.00)	< 0.01

Abbreviations: LAST, local anesthetic systemic toxicity; TPVB, thoracic paravertebral nerve block.

### 3.5. Postoperative Recovery Profiles

A statistical difference in postoperative hospital length of stay was observed between Groups S and D (*p* = 0.045). The median dose of postoperative rescue analgesic administration (tramadol) was lower in Group S (300 mg vs 400 mg). No significant differences were found between the two groups in terms of the number of effective PCIA attempts, total PCIA attempts, number of drainage tubes, duration of drainage tube placement, or time spent in the PACU (Table 4).

**TABLE 4:** Postoperative recovery profiles.

Indicator	Group D (*N* = 46)	Group S (*N* = 46)	*p*
Effective PCIA attempts, times/48h	0 (0, 2)	0 (0, 1)	0.325
Total PCIA attempts, times/48h	0 (0, 2)	0 (0, 1)	0.296
Tramadol consumption (mg)	400 (275, 825)	300 (175, 600)	0.054
Number of drainage tubes, *n* (%)			0.293
One tube	35 (76.1%)	39 (84.8%)	
Two tubes	11 (23.9%)	7 (15.2%)	
Drainage duration (days)	4.00 (3.00, 4.25)	4.00 (3.00, 4.00)	0.441
Hospital stay (days)	4.00 (4.00, 5.00)	4.00 (3.00, 4.25)	0.045
PACU time (min)	34.50 (28.50, 47.75)	35.00 (29.75, 55.75)	0.492

Abbreviations: PACU, postanesthesia care unit; PCIA, patient‐controlled intravenous analgesia.

## 4. Discussion

This study preliminarily assessed the comparative effectiveness of single‐injection versus dual‐injection of LB in TPVB for postoperative analgesia following dual‐microport thoracoscopic lobectomy. The most significant finding was a marked reduction in cumulative pain burden during the critical first 72 h postoperatively in the single‐injection group. This advantage was particularly pronounced at the 24‐h and 48‐h time points, which are typically associated with peak surgical pain and early mobilization. No episodes of severe pain were reported in Group S, whereas a significant incidence was observed in Group D, further underscoring the clinical significance of single‐injection therapy in preventing severe postoperative pain. Importantly, the simplified single‐injection technique substantially reduced procedural time and hospital stay, enhancing clinical efficiency without increasing complication rates.

During the first 72 h postoperatively, the incidence of moderate pain was similar between Groups S and D, both at rest and during movement. In contrast, no patients in Group S reported severe pain at any time during the observation period. While the AUC at rest did not reach statistical significance, Group S showed a 19.7% reduction in pain compared to Group D, suggesting a potential but nonsignificant analgesic benefit. Overall, Group S provided better analgesia within the first 72 h postoperatively, resulting in a lower cumulative pain burden for patients. From a mechanism perspective, a single injection of LB achieves a higher local concentration due to the liposomal sustained‐release carrier, which slowly releases the drug within the tissue, allowing it to diffuse and cover multiple intercostal nerves. In contrast, while dual‐injection provides more direct and broader initial coverage, the fixed total volume may lead to a 30%–50% reduction in peak local tissue concentration [18]. Previous anatomical studies have shown that a single 20 mL thoracic paravertebral injection of dye at thoracic vertebrae 4‐5 spreads longitudinally over 5.0 ± 1.1 segments, with diffusion in both cranial and caudal directions [19]. The surgical field for dual‐microport thoracoscopic lobectomy primarily involves thoracic vertebrae 3–7. This anatomical coverage, combined with the extensive caudal‐cranial diffusion achieved by a single 20 mL LB injection at thoracic vertebrae 4‐5, ensures adequate drug coverage throughout the surgical area. Notably, no significant difference in analgesic effect was observed between the two groups at the 6‐h and 72‐h time points. This may be because LB requires over 6 h to achieve steady‐state release, with early analgesia depending on the initial free bupivacaine. The full extent of the blockade has not yet been established [20]. The duration of LB’s action is approximately 72 h [21], at which point drug release in both groups enters a decline phase, leading to natural pain resolution. Additionally, the 3‐month postoperative follow‐up showed no significant differences in the incidence of CPSP between the two groups. This may be attributed to LB’s sustained‐release properties, which provided continuous analgesia during the acute pain phase, effectively reducing hyperalgesia and subsequently lowering the risk of CPSP. A randomized controlled trial demonstrated that thoracoscopic lobectomy patients receiving TPVB with LB had a 10.5% incidence of moderate‐to‐severe CPSP at 3 months postoperatively, representing a significant 43% reduction compared to the bupivacaine group [22].

The results of this study demonstrate that single‐injection blockade effectively reduced pain scores during the 24–48 h postoperative period, shortened hospital length of stay, and decreased TPVB procedure duration. The observed difference in hospital stay, though only one day in median values, was statistically significant and may be clinically relevant. In Group D, a higher proportion of patients required two chest tubes (23.9% vs. 15.2%) and had numerically greater rescue analgesic consumption, which could delay achievement of discharge criteria, such as chest tube removal and pain control during movement. All discharges followed a standardized postoperative pathway, and no early discharge for nonmedical reasons occurred in either group. Some studies have suggested that the intensity of pain control within the first 48 h after thoracoscopic lobectomy is negatively correlated with the quality of postoperative recovery [23]. In contrast, the dual‐injection blockade technique did not show superiority in this study. Although dual‐injection theoretically offers more comprehensive analgesia through a broader coverage area, our data did not reveal any additional clinical benefits over single‐injection blockade in reducing postoperative pain scores, decreasing opioid consumption, or shortening hospital stay. These findings align with existing literature. A randomized controlled trial in lumbar discectomy found no significant differences between single‐injection and dual‐injection erector spinae plane blocks regarding NRS scores, opioid consumption, or length of hospital stay within the first 24 h postoperatively [24]. Furthermore, other studies have indicated that while a dual‐injection technique with ropivacaine in ESPB or TPVB may yield a slightly faster onset and higher block level compared to a single injection, it does not provide superior postoperative analgesic effects. Additionally, the dual‐injection approach requires an extra puncture, prolongs procedure time, and may compromise patient comfort [25, 26]. Collectively, these results suggest that for the surgical type and patient population involved in this study, adding a second blockade point did not lead to the anticipated clinical benefits. Notably, LB’s liposomal technology significantly reduces the risk of acute systemic toxicity. Its peak plasma concentration is lower than that of traditional bupivacaine, and no serious adverse reactions were observed in this study [27], further supporting the clinical safety of single‐injection TPVB.

This study has several limitations. First, the relatively small sample size may have limited the statistical power for secondary outcome measures, such as the incidence of CPSP and pulmonary complications. Future research could expand the sample size or adopt a multicenter design to improve statistical power and external validity. Second, as nerve blocks were performed after the induction of general anesthesia, which effectively prevents patient discomfort or movement from interfering with the procedure, a limitation is the inability to objectively assess the onset time of the blockade and the extent of the sensory block. Third, due to inherent technical differences between the two blockade techniques, blinding of the physicians performing the procedures was not feasible, which may have introduced performance bias. However, its impact on the primary outcome is likely minimal, as both pain assessors and patients were blinded, effectively preventing detection bias, and all perioperative management followed standardized protocols. Finally, the lack of objective pain‐related metrics is a limitation; future studies could incorporate objective tools, such as dynamic pain monitoring devices or tracking changes in inflammatory cytokine concentrations, to reduce subjective bias.

## 5. Conclusion

This study suggests that single‐injection nerve block techniques may be considered a safe, effective, and operationally simplified preferred option when formulating postoperative analgesia plans for patients undergoing double‐port thoracoscopic lobectomy. Specifically, the use of LB in single‐injection TPVB not only ensures effective pain control but also significantly reduces the incidence of postoperative complications, facilitating enhanced recovery. In contrast, double‐injection nerve block techniques did not demonstrate superior analgesic efficacy in this study, and their operational complexity may introduce potential risks. Of course, the conclusions of this study stem from a small sample randomized controlled trial, and their generalizability requires further validation through larger‐scale research. Future research should focus on refining procedural standards and efficacy evaluation systems for single‐injection nerve block techniques, as well as identifying specific patient populations or surgical types that may benefit from more complex block strategies. These efforts will help advance postoperative pain management toward a more precise and individualized approach.

**Nomenclature**
TPVB: Thoracic paravertebral nerve block
LB: Liposome bupivacaine
CPSP: Chronic postsurgical pain
NRS: Numerical rating scale
PCIA: Patient‐controlled intravenous analgesia
AUC: Area under the curve
ASA: American Society of Anesthesiologists
BMI: Body mass index
CI: Confidence interval
PACU: Postanesthesia care unit
SD: Standard deviation
IQR: Interquartile range
GEE: Generalized estimating equations
CT: Computed tomography

## Author Contributions

Experimental design: Yue Shang and Changjun Gao. Nerve block interventions: Li Sun and Jiameng Wang. Postoperative follow‐up: Wenlun Zhong and Chenyang Wang. Data collection and processing: Yue Shang, Wenlun Zhong, and Jiameng Wang. Drafted manuscript: Yue Shang. Edited manuscript: Yue Shang and Changjun Gao.

## Funding

This study was funded by the Clinical Research Project of the Medical and Health Science and Technology Development Research Center of the National Health Commission: WKZX2024CX301202.

## Ethics Statement

This prospective randomized, double‐blind, controlled trial was approved by the Ethics Committee of Tangdu Hospital, Fourth Military Medical University (approval number: K202506‐14).

## Consent

All authors have consented to the publication of this study.

## Conflicts of Interest

The authors declare no conflicts of interest.

## Supporting Information

Additional supporting information can be found online in the Supporting Information section.

## Supporting information


**Supporting Information** The Supporting file contains the CONSORT 2025 checklist for this randomized controlled trial. It details the itemized adherence to the CONSORT statement.

## Data Availability

The datasets used and analyzed during the current study are available from the corresponding author upon reasonable request. Requests may require approval from an ethics committee or institutional review board. Raw clinical data are not publicly available due to patient privacy restrictions but may be shared in anonymized form under a data transfer agreement.
